# Novel imidazotetrazine derivatives overcome temozolomide resistance in glioblastoma by inducing ferroptosis and apoptosis

**DOI:** 10.1038/s41420-025-02857-3

**Published:** 2026-01-09

**Authors:** Heng Yang, Wei Zhao, Yutao Huang, Yan Wu, Yongdong Zou, Ting Wang, Lizhi Zhu, Baomin Xi, Duo Zheng

**Affiliations:** 1 Guangdong Provincial Key Laboratory of Genome Stability and Disease Prevention, Shenzhen Key Laboratory of Translational Medicine in Oncology, International Cancer Center, School of Basic Medical Sciences; School of Pharmaceutical Sciences, Medical School; College of Life Sciences and Oceanography, Shenzhen University, Shenzhen, Guangdong China; 2 Guangzhou KemRocMed Co. Ltd., Guangzhou, China; 3 Shenzhen Zhonghe Headway Bio-Sci & Tech Co., Ltd., Shenzhen, Guangdong China; 4 Guangdong Provincial Key Laboratory of New Drug Screening, Guangzhou Key Laboratory of Drug Research for Emerging Virus Prevention and Treatment, School of Pharmaceutical Sciences, Southern Medical University, Guangzhou, China

**Keywords:** CNS cancer, Pharmacodynamics, Drug discovery and development, Toxicology, Drug delivery

## Abstract

Glioblastomas (GBM) is a highly malignant primary brain tumor with poor prognosis despite standard treatments of surgery, radiotherapy, and chemotherapy. Temozolomide (TMZ) is a key chemotherapeutic agent for GBM but is limited by resistance mechanisms. In this study, we developed a novel imidazotetrazine analogs to overcome TMZ resistance with enhanced therapeutic efficacy. CCK8 assays demonstrated that QX302 showed remarkable potency and effectively inhibited the viability of U251, U87, T98G, and HCT116 cells in a dose- and time-dependent manner. Proteomic analysis indicated that QX302 affected critical pathways, including nucleotide binding, chromatin organization, cell cycle regulation, and DNA repair processes. Further investigations revealed that QX302 effectively inhibits glioma spheroid growth and induces cell cycle arrest, ferroptosis, and apoptosis. Notably, QX302 induced DNA damage in cancer cells via the alkylation of DNA, leading to increased sensitivity to Olaparib via the base excision repair signaling pathway. Predictive modeling demonstrated QX302 has a favorable pharmacokinetic profile, including high blood-brain barrier permeability, highlighting its potential as a central nervous system-penetrating therapeutic agent. In conclusion, QX302 represents a promising therapeutic strategy for GBM, offering improved efficacy and the potential for use in combinatorial therapy with lower effective doses compared to TMZ.

## Introduction

A glioblastoma (GBM) is a primary brain tumor of the central nervous system (CNS) characterized by a high mortality rate and median survival of 15 months after the initial diagnosis [1]. In the past decade, incidences of CNS tumors have steadily increased, reaching 10 per 100,000 people [2]. The cornerstone of GBM treatment is surgery, often followed by radiotherapy and adjuvant chemotherapy [1]. However, despite these treatment modalities, the prognosis for GBM remains poor due to several factors. The infiltrative nature of the tumor often results in incomplete surgical resection, leaving behind residual cancer cells that contribute to recurrence [3]. Additionally, the blood-brain barrier (BBB) limits the effectiveness of systemic chemotherapy agents, reducing their ability to reach the tumor site in adequate concentrations [4]. GBM cells can also develop resistance to radiation and chemotherapy over time, further complicating treatment outcomes [5]. These challenges underscore the critical need for continued research into new therapeutic strategies that can target the unique biology of GBM and improve patient outcomes.

Temozolomide (TMZ) is an oral alkylating agent utilized in the treatment of CNS tumors. It possesses several favorable characteristics, including high bioavailability, significant fat solubility, ability to easily cross the BBB, potent efficacy, and mild bone-marrow inhibition [6]. Mechanistically, TMZ primarily functions by alkylating purine bases, specifically methylating the N7 of guanine, the N3 of adenine, and the O6 of guanine [7, 8]. This alkylation induces nucleotide mismatching during subsequent replication cycles [9, 10]. Repair mechanisms such as O6-methylguanine-DNA methyltransferase (MGMT), mismatch repair (MMR), and base excision repair (BER) work to correct TMZ-induced DNA adducts [11]. MMR, in particular, generates DNA double-strand breaks in TMZ-sensitive cells, ultimately triggering programmed cell death [12]. However, the elevated expression of MGMT facilitates the removal of TMZ adducts, resulting in TMZ-resistant glioma cells [13].

Research advances have been made in the development of TMZ analogs and imidazotetrazine alkylating agents for overcoming TMZ-resistance mechanisms in cancer therapy, with studies focusing on enhancing their bioavailability and BBB-penetration capacity. Riley et al. [14] designed a novel compound, CPZ, by introducing an acetylene group at the N3 position and a chlorine atom at the C8 position of TMZ. This modification bypasses the MGMT-mediated repair mechanism and significantly enhances the hydrolysis stability of CPZ, improving its activity and bioavailability in vivo. Another study discovered that modifications of TMZ, specifically C8-imidazole (377) and C8-methylimidazole (465) at the carboxylamide group, enhanced compound stability and anti-tumor efficacy. These modifications were effective in mitigating the drug resistance mediated by MGMT overexpression and MMR deficiency [15].

In structural modification studies, researchers aiming to optimize treatment outcomes have also explored the development of novel TMZ analogs with improved pharmacokinetic profiles and enhanced tumor-penetration properties. Yang et al. [16] developed two novel TMZ analogs, DP68 and DP86, that feature a p-methyl-substituted pyrazine ring. DP68 serves as a precursor to the aziridinium ion generated by nitrogen mustard drugs, altering the chemical mechanism to circumvent known TMZ-resistance mechanisms. Importantly, DP68 exerts therapeutic effects by targeting the primary reaction site N7-guanine, rather than the secondary site O6-guanine. Another effort included the design of prodrugs that release active alkylating agents selectively within tumor tissues, as well as dual-targeting strategies to maximize the therapeutic impact [17, 18]. Although the aforementioned new TMZ analogs exhibit excellent antitumor activity, their long-term toxicity, impact on normal cells, and potential side effects have not been thoroughly studied. In particular, the potential resistance mechanisms, including changes to DNA repair pathways, the tumor microenvironment, and the extracellular matrix, have yet to be fully explored.

In clinical settings, chlorambucil is a frequently employed nitrogen mustard alkylating agent [19]. The use of this agent in combination therapies with TMZ has been documented [20, 21]. In this study, chlorambucil was conjugated to an imidazotetrazine group through ester and amide bonds to synthesize the imidazotetrazine compounds QX302 with the aim of showcasing their improved antitumor efficacy. The anti-glioma activity test found that QX302 exerted a substantial inhibitory effect on glioma cells in vitro. Specifically, QX302 exhibited significant anticancer efficacy against MGMT-overexpressing glioma T98G and MMR-deficient colorectal carcinoma HCT116 cell lines. QX302 demonstrated the ability to halt cell cycle progression at the G2/M phase, induce DNA damage, and instigate apoptosis and ferroptosis in GBM cells, regardless of their MGMT and MMR expression status. Overall, our results support the validity of further investigations into QX302, a novel therapeutic agent for glioma treatment with outcomes superior to those of current standard therapies, to provide new avenues for the treatment of patients facing TMZ resistance.

## Results

### QX302 exerts a significant anti-glioma effect in vitro

**Fig. 1:**
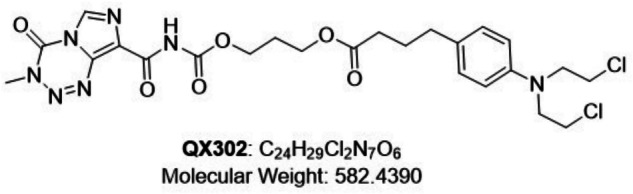
Chemical structural formulae of QX302.

To address the limitations of TMZ’s efficacy and further enhance its therapeutic effects, extensive structural modifications were carried out using TMZ as the base compound. We modified the bicyclic structure of imidazotetrazine, specifically with substitutions on the C8 with chlorambucil. This modification led to the synthesis of QX302 (Fig. 1). Subsequently, we utilized a CCK8 assay to assess the impact of QX302 on the viability of various glioma cell lines. The results demonstrated the clear inhibition of cell viability by QX302 in a dose- and time-dependent manner (Table 1). Remarkably, QX302 exhibited the highest cytotoxicity in the U251 cell line, suggesting it has a strong anti-proliferative effect on this particular cell type. Its toxicity was lower in the T98G cell line, which is known for its high MGMT expression, indicating a potential resistance mechanism in these cells (Table 1). To further explore this phenomenon, we investigated the effect of QX302 on HCT116 cells, another MGMT+/MMR− cell line (Table 1). Our findings revealed that the toxicity of QX302 was less pronounced in HCT116 cells than in U251 and U87 cells, reinforcing the conclusion that MGMT expression influenced the cells’ sensitivity to QX302. Moreover, the cytotoxic effect of QX302 was significantly greater at 48 h than at 24 h, highlighting the increased effectiveness of the compound with extended exposure duration. These observations underscore the potential of QX302 as a concentration- and time-dependent therapeutic agent against glioma cells.

**Table 1:** CCK8 assay IC50 values of QX302 against tumor cell lines.

IC50 (μM)	24 h	48 h	72 h
Time			
U251	108.0 ± 7.40	9.229 ± 0.42	7.787 ± 0.32
U87	109.2 ± 10.3	23.46 ± 1.07	18.44 ± 0.91
T98G	196.4 ± 12.6	96.04 ± 5.97	86.13 ± 10.6
HCT116	197.7 ± 42.8	55.69 ± 9.55	24.80 ± 4.70

CCK8 assays were performed on U251, U87, T98G, and HCT116 cell lines treated with QX302 for varying durations (24, 48, and 72 h) to generate IC50 curves.

**Fig. 2:**
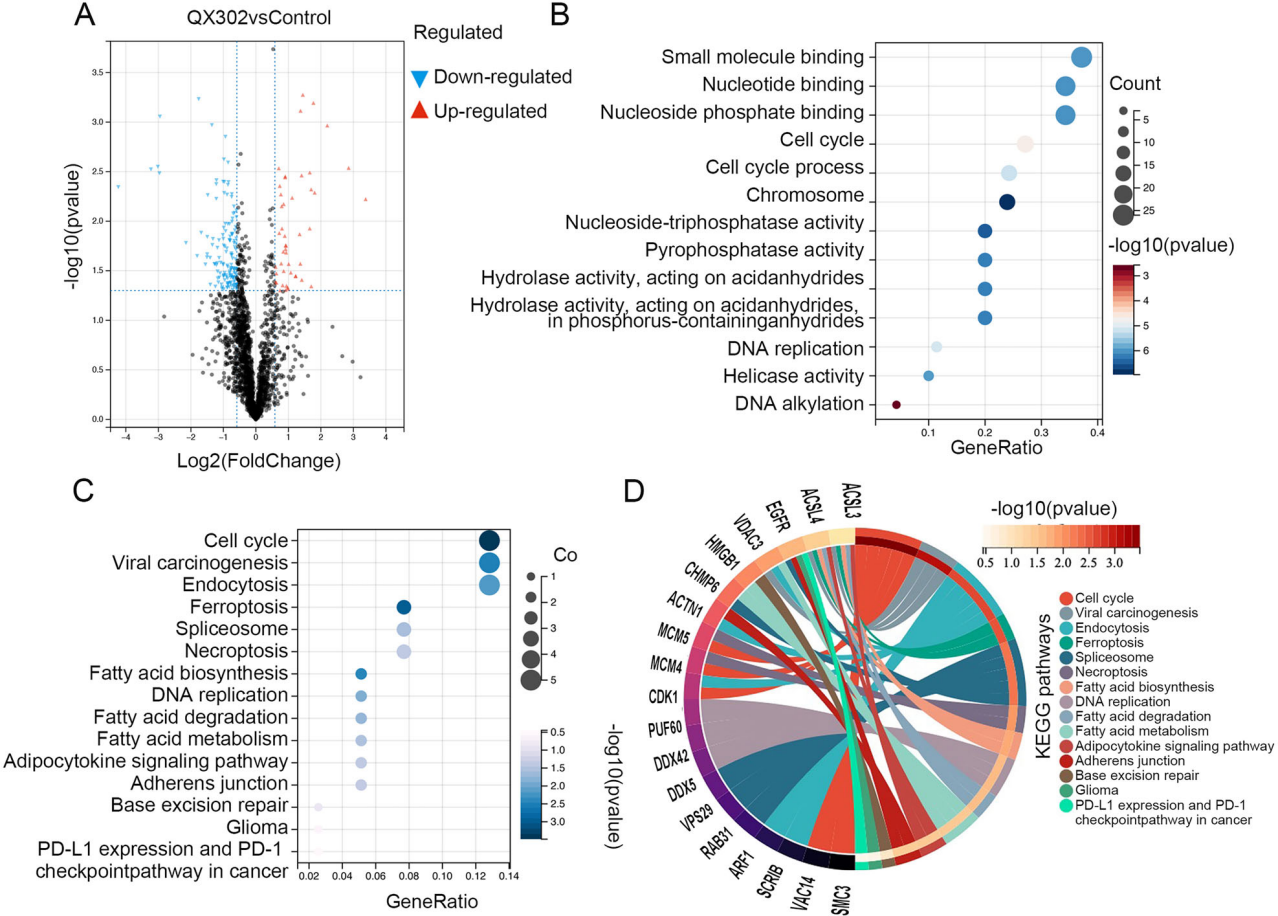
Effects of QX302 on proteomics of glioma cells. **A** Volcano map illustrating the differential expression of proteins in U251 cells following 72 h treatment with 10 µM QX302 compared to that of control group treated with DMSO. The x and y axes of the map represent the log2(FC) and -log10(pvalue), respectively. Black dots indicate genes that did not show significant changes, while blue and red dots represent significantly downregulated and upregulated genes, respectively. GO enrichment analysis (**B**) and KEGG pathway enrichment (**C**, **D**) analyses of the differential proteins.

U251 cells were treated with QX302 (10 μM) for 72 h, and comprehensive whole-cell proteomics analysis was conducted using LC-MS/MS. The differential protein data were screened using a fold change (FC) of ≤0.67 or ≥1.5 and *P* < 0.05, identifying a total of 146 significantly downregulated proteins and 47 significantly upregulated proteins (Fig. 2A). GO enrichment analysis of these differential proteins revealed significant enrichment in several critical categories, including small molecule binding, nucleotide binding, chromatin organization, cell cycle regulation, DNA replication, and DNA alkylation (Fig. 2B). These findings suggest that QX302 profoundly impacts various cellular functions at the molecular level. Furthermore, KEGG enrichment analysis (Fig. 2C, D) indicated that the differentially expressed proteins are predominantly associated with key biological pathways, particularly those involved in the cell cycle, ferroptosis, necroptosis, DNA replication, and DNA damage repair. These pathways are crucial for maintaining cellular integrity and responding to genotoxic stress, underscoring the potential mechanism through which QX302 exerts its cytotoxic effects. Collectively, these analyses provided us a detailed understanding of the molecular alterations induced by QX302 and highlight its potential impact on critical cellular processes in glioma cells.

### QX302 inhibits glioma cell proliferation by inducing G2/M cell cycle arrest

**Fig. 3:**
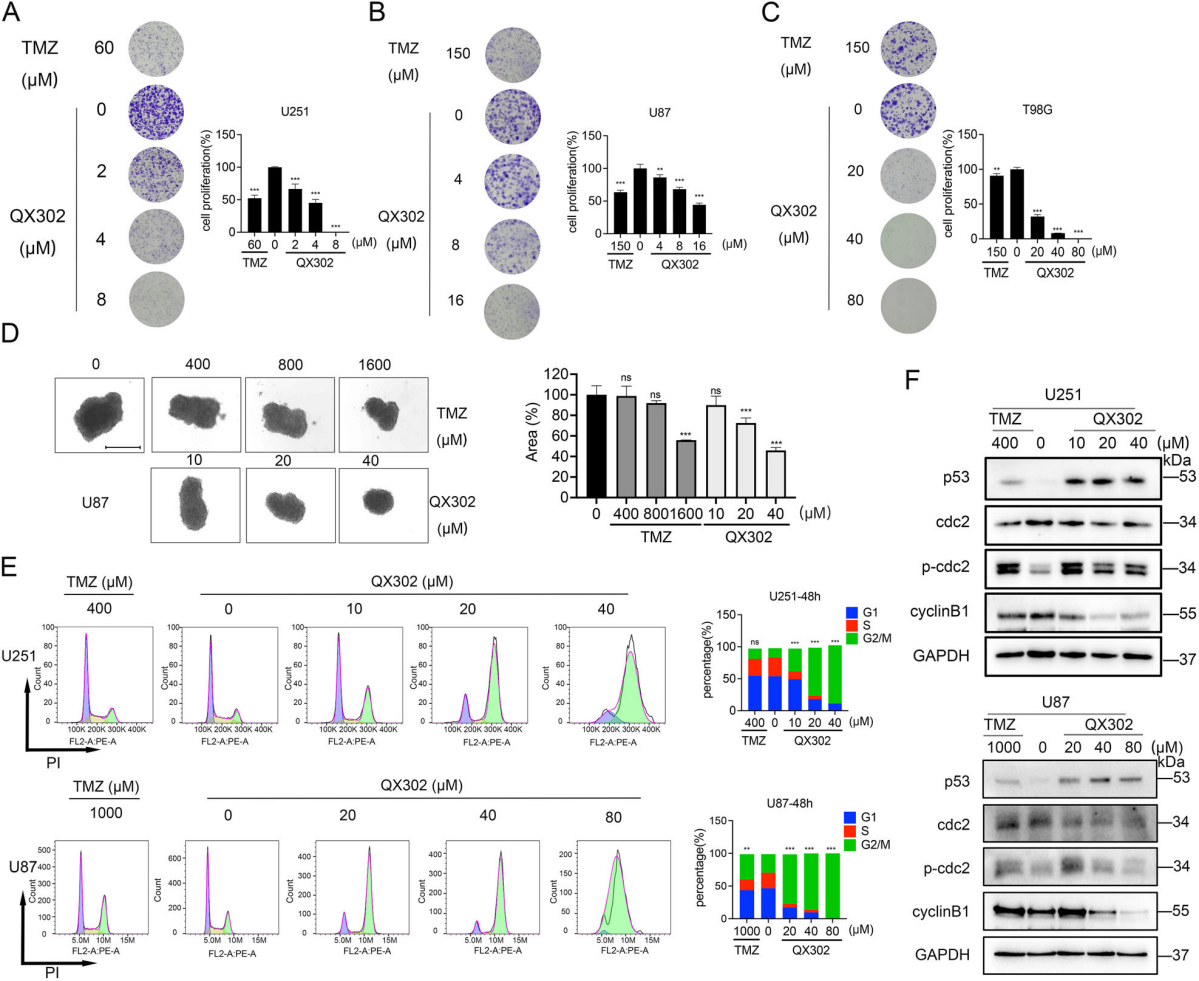
QX302 suppresses glioma cell proliferation by inducing G2/M cell cycle arrest. The effect of QX302 on glioma cell proliferation was assessed using a plate colony formation assay. U251 cells (**A**) were treated with 2, 4, and 8 μM QX302; U87 cells (**B**) were treated with 4, 8, and 16 μM QX302; and T98G cells (**C**) were treated with 20, 40, and 80 μM QX302. TMZ served as the positive control. After 10–14 days, cells were stained with crystal violet. **D** The effect of QX302 on the growth of U87 cell spheres was analyzed (Scale bars = 500 μm). U87 cell spheres were treated with 10, 20, and 40 μM QX302, while TMZ-treated cells received 400, 800, and 1600 μM. Six days later, cells were stained and photographed for analysis. **E** Representative images of cell cycle distribution measured by flow cytometry. U251 and U87 cells were treated with 10, 20, and 40 μM QX302 for 48 h, and 20, 40, and 80 μM QX302 for 48 h, respectively. DMSO was used as the negative control and TMZ as the positive control. The histograms on the right display the percentage of cells in the G0/1 (left peak), S (middle peak), and G2/M (right peak) phases for each treatment group. **F** Western blot analysis of U251 cells treated with 10, 20, and 40 μM QX302 for 72 h, and U87 cells treated with 20, 40, and 80 μM QX302 for 48 h. DMSO was the negative control and TMZ the positive control. The expression levels of P53, CDC2, p-CDC2, CyclinB1, and GAPDH (internal control) were assessed. The data shown represent the means ± SD from three independent experiments. ns > 0.05, *p < 0.05, **p < 0.01 and ***p < 0.001, compared to control group.

Next, we found that QX302 significantly inhibited the proliferation of glioma cells at lower concentrations than the positive-control drug TMZ, as evidenced by the cell plate cloning assay (Fig. 3A–C). This inhibition of glioma cell proliferation by QX302 is consistent with previous observations of its cytotoxicity towards glioma cells. The tumor cell sphere model, which accurately simulates the in vivo growth of glioma cells, was used to further assess the effects of QX302. The 3D sphere-forming assay demonstrated that QX302 at 40 μM had a significantly stronger inhibitory effect (*P* < 0.001) on the spherical growth of U87 cells than TMZ at 1600 μM (Fig. 3D). Additionally, at a concentration of 20 μM, QX302 caused a notable reduction (*P* < 0.001) in sphere volume relative to the control group. In addition, previous KEGG enrichment analysis suggested that QX302 exerted its anti-proliferative effects by influencing the cell cycle of glioma cells. These findings collectively highlight the potent anti-proliferative properties of QX302 in glioma cells and suggest it has potential to be an effective therapeutic agent. The lower effective concentration of QX302 compared to TMZ underscores its efficacy and potential for reduced side effects in therapeutic applications.

Flow cytometry analysis revealed that QX302 significantly arrested the cell cycle of glioma cells (Fig. 3E). Compared to the control group, there was a concentration-dependent increase in G2/M phase cells and decrease in S and G1 phase cells [22, 23]. Notably, the cell cycle arrest induced by QX302 was more pronounced than that induced by TMZ, with the most substantial arrest observed after 48 h of treatment. This suggests that QX302 is more effective at inducing cell cycle arrest in glioma cells. The degradation of cyclin B1 during mitosis is a critical step for proper cellular mitosis [24, 25]. In U251 and U87 cells, QX302 treatment substantially upregulated the protein levels of p53 and phosphorylated cdc2, while markedly decreasing the protein level of cyclin B1 (Fig. 3F). This reduction in cyclin B1 became more pronounced with increasing doses of QX302, indicating that more cells were exiting mitosis. These results collectively suggest that QX302 induces cell cycle arrest in a dose-dependent manner, potentially through the modulation of key regulatory proteins involved in mitosis.

### QX302 promotes GBM cells ferroptosis by inducing ROS generation, Fe^2+^ accumulation, and lipid peroxidation

**Fig. 4:**
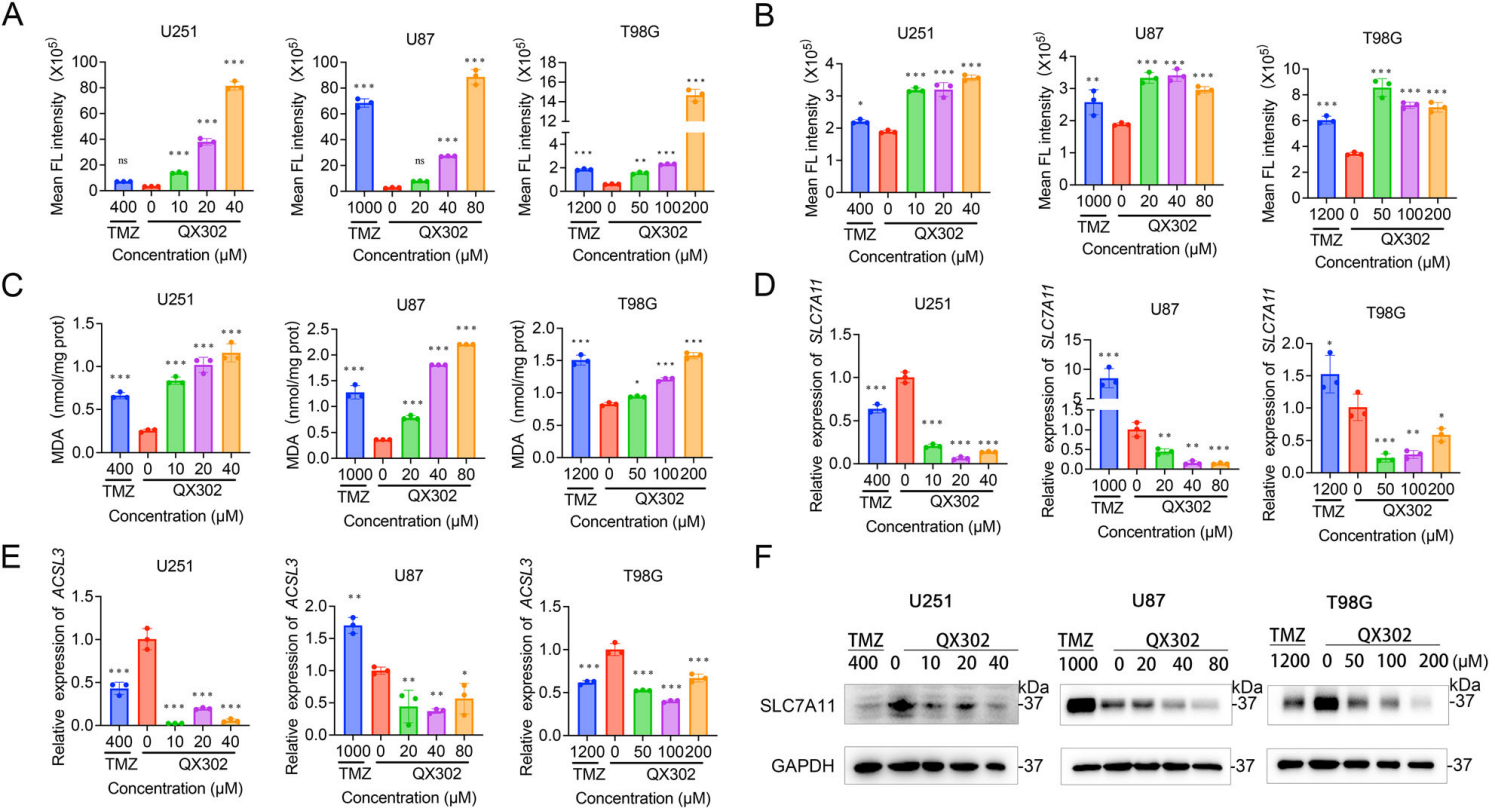
QX302 induces ferroptosis in glioma cells by inducing ROS generation, Fe^2+^ accumulation, and lipid peroxidation. **A** Flow cytometry was used to evaluate the ROS generation of U251, U87 and T98G cells after treated with various concentrations of QX302; TMZ-treated cells were used as controls. **B** Intracellular Fe^2+^ content was assessed by flow cytometry in U251, U87, and T98G cells after 72 h of treatment with QX302 or TMZ. **C** Intracellular MDA content was measured by MDA kit after 72 h of QX302 or TMZ treatment in U251, U87, and T98G cells. **D**, **E** The mRNA levels of SLC7A11 and ACSL3 in U251, U87, and T98G cells treated with QX302 or TMZ for 48 h. **F** Protein expression of SLC7A11 was detected by western blotting in GBM cell lines treated with QX302 or TMZ at the indicated concentrations for 72 h. The data shown represent the means ± SD from three independent experiments. ns > 0.05, *p < 0.05, **p < 0.01 and ***p < 0.001, compared to control group.

To assess ferroptosis induction, we first evaluated intracellular levels of ROS and Fe²⁺ using the fluorescent probes DCFH-DA and FerroOrange, respectively. Flow cytometry analysis revealed that treatment with varying concentrations of QX302 or TMZ for 72 h significantly increased both ROS (Fig. 4A) and Fe^2⁺^ levels (Fig. 4B) in U251, U87, and T98G cell lines, indicating that QX302, like TMZ, induces oxidative stress and promotes ferroptosis. Additionally, we measured MDA content in these cell lines following treatment with QX302 or TMZ. As shown in Fig. 4C, both QX302 and TMZ significantly increased MDA levels in U251, U87, and T98G cells, confirming the promotion of lipid peroxidation. To explore the molecular mechanisms underlying ferroptosis induction by QX302, we assessed the expression of the SLC7A11 and ACSL3 genes using qPCR. The results showed that QX302 treatment significantly downregulated the expression of both SLC7A11 and ACSL3 in U251, U87, and T98G cells (Fig. 4D, E). Western blot analysis further confirmed that QX302 significantly reduced SLC7A11 protein levels in U251, U87, and T98G cells (Fig. 4F), indicating a strong induction of ferroptosis across different GBM cell lines. In contrast, the expression of SLC7A11 and ACSL3 was not reduced in TMZ-treated U87 cells, suggesting a differential mechanism between QX302 and TMZ. These findings suggest that QX302 induces ferroptosis in GBM cells by regulating key factors such as SLC7A11, with a more pronounced effect compared to TMZ.

### QX302 induces apoptosis of glioma cells

**Fig. 5:**
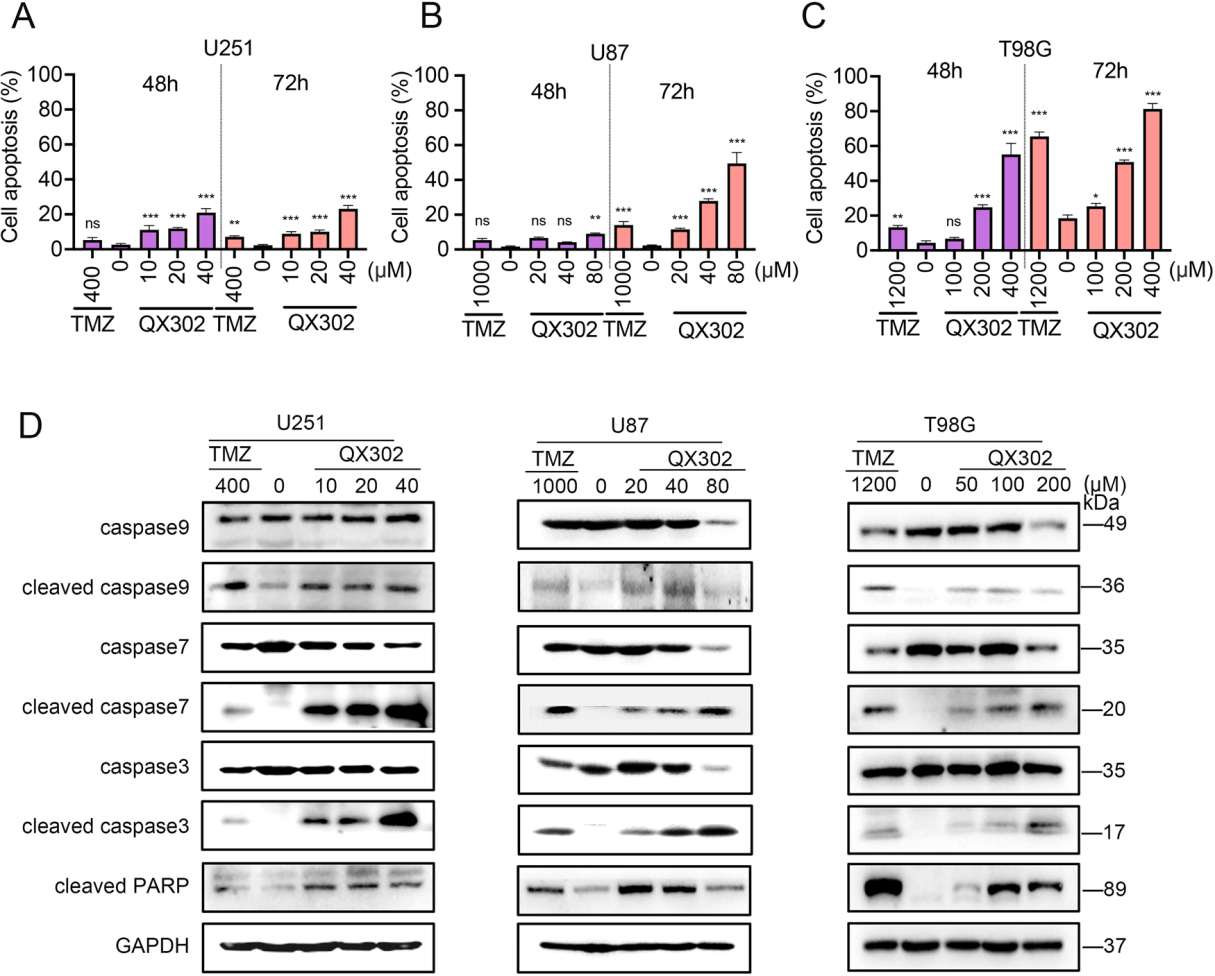
QX302 induces apoptosis in glioma cells. **A**–**C** Histograms of cell apoptosis detected by flow cytometry. U251 cells were treated with 10, 20, and 40 μM QX302, U87 cells with 20, 40, and 80 μM QX302, and T98G cells with 100, 200, and 400 μM QX302 for 48 and 72 h. DMSO was used as the negative control and TMZ as the positive control. **D** Western blot analysis of U251 cells treated with 10, 20, and 40 μM QX302, U87 cells treated with 20, 40, and 80 μM QX302, and T98G cells treated with 50, 100, and 200 μM QX302 for 72 h. DMSO served as the negative control and TMZ as the positive control. Protein expression levels of Caspase 9, Cleaved Caspase 9, Caspase 7, Cleaved Caspase 7, Caspase 3, Cleaved Caspase 3, Cleaved PARP, and GAPDH (internal reference) were detected.The data shown represent the means ± SD from three independent experiments. ns > 0.05, *p < 0.05, **p < 0.01 and ***p < 0.001, compared to control group.

Furthermore, flow cytometry showed that QX302 induced apoptosis in glioma cells (Fig. 5A–C). The apoptotic effect of QX302 on glioma cells was stronger and achieved at lower concentrations than that in both the control group and TMZ group. Apoptosis was significantly more evident after 72 h of treatment compared to 48 h. The increased occupancy of cells in the G2/M phase coincided with a higher number of apoptotic cells. In U251, U87, and T98G cells, the protein levels of cleaved caspase-9, cleaved caspase-7, cleaved caspase-3, and cleaved PARP were considerably elevated after 72 h of QX302 treatment (Fig. 5D). The presence of cleaved caspase-3 and cleaved PARP serves as an indicator of apoptotic cell death. These findings indicate that QX302 induced apoptosis in glioma cells in a concentration- and time-dependent manner. The ability of QX302 to effectively trigger apoptosis at lower concentrations than TMZ underscores its potential for use as a potent therapeutic agent against glioma.

### QX302 induces DNA damage in glioma cells by alkylating DNA

**Fig. 6:**
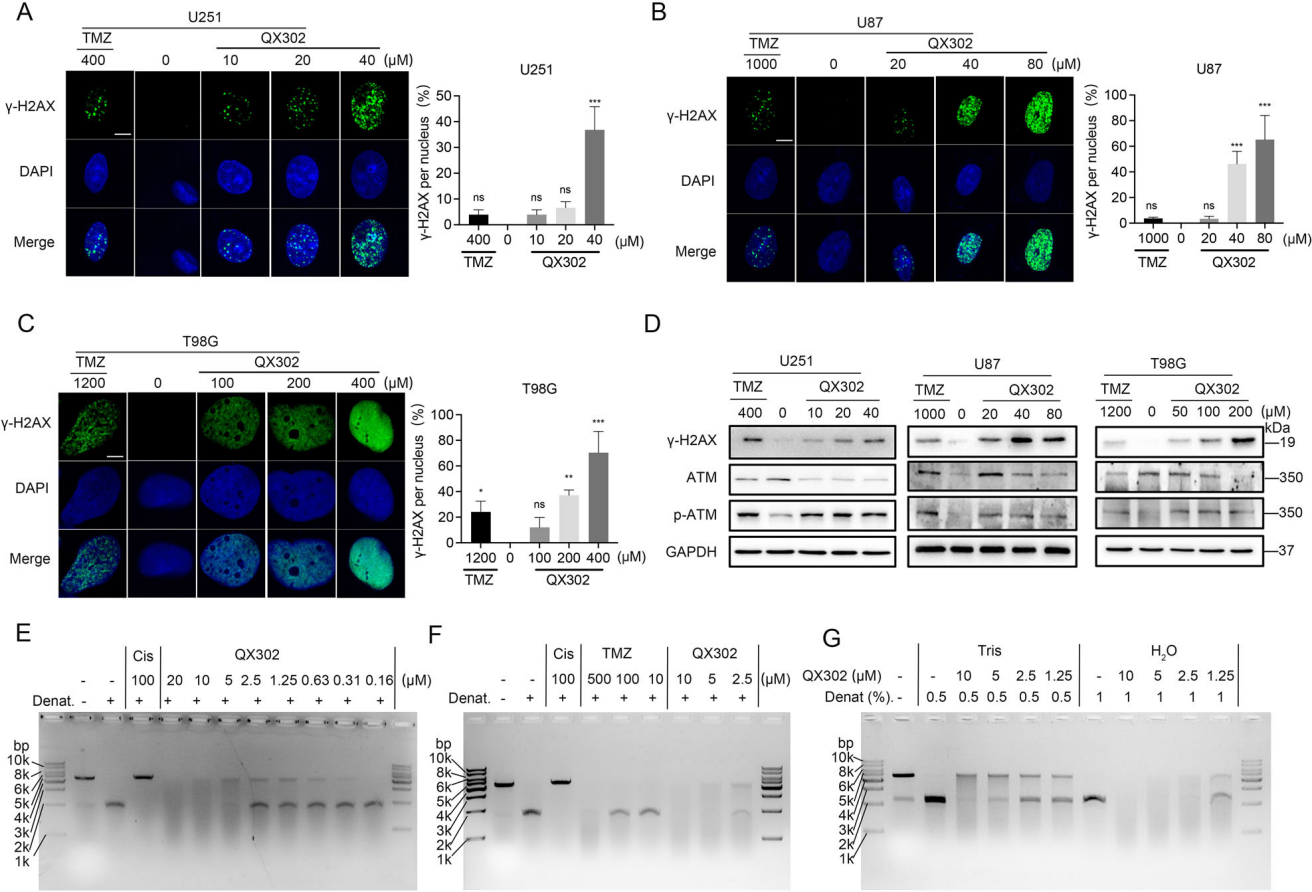
QX302 induces DNA damage in glioma cells through alkylation-like modification. Immunofluorescence staining for γH2AX was performed on U251 cells (**A**) treated with 10, 20, and 40 μM QX302, U87 cells (**B**) treated with 20, 40, and 80 μM QX302, and T98G cells (**C**) treated with 50, 100, and 200 μM QX302 for 72 h.DMSO was used as the negative control and TMZ as the positive control. The accompanying graphs show the statistical analysis of the proportion of γH2AX-positive nuclei. **D** Western blot analysis of ATM, p-ATM, γH2AX, and GAPDH (internal reference) protein levels in U251, U87, and T98G cells treated with QX302 for 72 h. **E** Linearized pUC19 plasmid DNA (100 ng) was treated with various concentrations of QX302 at 37 °C for 15 h, followed by denaturing gel electrophoresis with cisplatin as the control. **F** The effect of QX302 and TMZ (positive control) on plasmid DNA under different denaturing conditions. **G** The effect of QX302 on DNA alkylation when assayed under different experimental conditions. Tris = Tris buffer (pH 8.5); H_2_O = unbuffered water (pH 7); 0.5/1 = 0.5% or 1% NaOH were used to denature pUC19 plasmid DNA. The data shown represent the means ± SD from three independent experiments. ns > 0.05, *p < 0.05, **p < 0.01 and ***p < 0.001, compared to control group.

Proteomic analysis indicated that QX302 influences DNA replication and repair mechanisms. To further investigate this, we assessed the levels of the DNA damage marker γ-H2AX with an immunofluorescence assay. The results demonstrated that QX302 treatment significantly elevated γ-H2AX levels in the nuclei of glioma cells compared to the control and TMZ treatments (Fig. 6A–C). Additionally, the protein levels of both γ-H2AX and phosphorylated ATM (p-ATM) were dramatically upregulated in a QX302-dose-dependent manner (Fig. 6D). An in vitro alkylation assay revealed that the extent of DNA alkylation by QX302 was concentration-dependent (Fig. 6E). Remarkably, QX302 exhibited more potent DNA cross-linking capabilities at lower concentrations than the cross-linking agent cisplatin, and at higher concentrations, the excessive alkylation of DNA led to its degradation (Fig. 6E). Notably, QX302 at 5 μM alkylated DNA more effectively than TMZ at 500 μM (Fig. 6F). Furthermore, variations in solvent conditions did not affect QX302’s ability to cross-link and alkylate DNA (Fig. 6G). These findings suggest that QX302 is a highly effective DNA-alkylating agent capable of inducing significant DNA damage at lower concentrations.

### QX302 increases the sensitivity of glioma cells to Olaparib through the BER signaling pathway

**Fig. 7:**
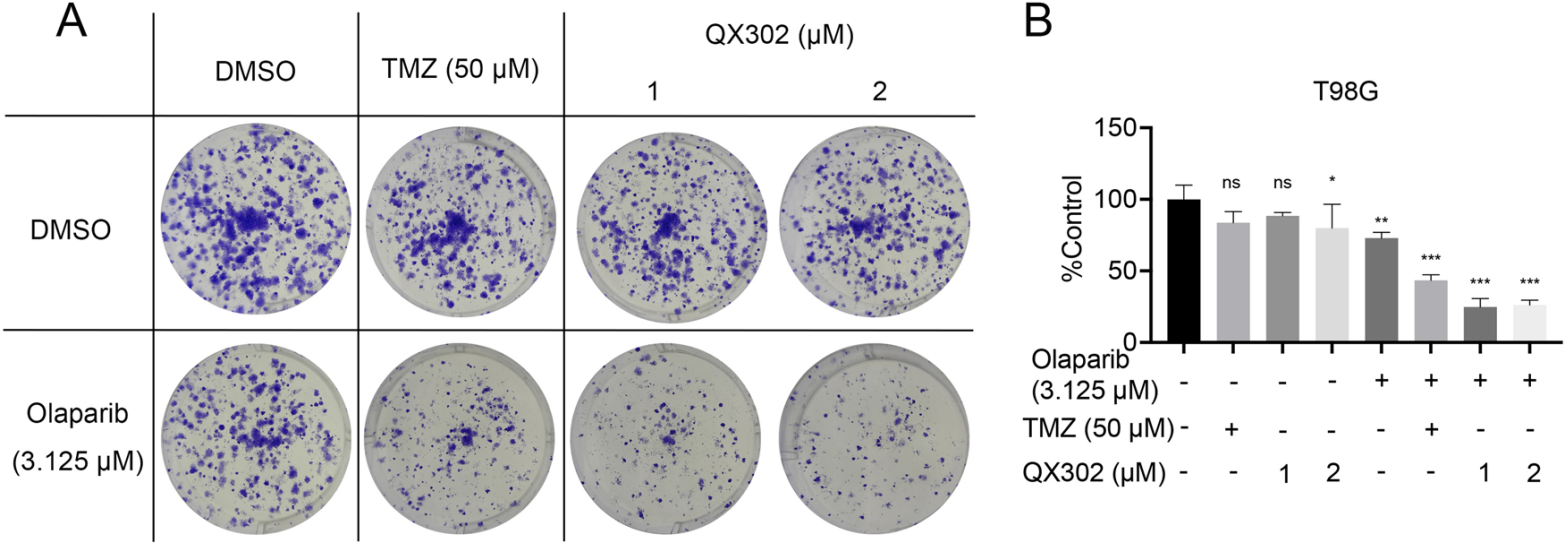
QX302 increases the sensitivity of glioma cells to olaparib via the BER signaling pathway. **A** Survival of T98G cells treated with 3.125 μM olaparib or DMSO (negative control) followed by treatment with 1 μM or 2 μM QX302. Cells treated with 50 μM TMZ alone or in combination with 3.125 μM olaparib served as controls. Cells were stained with crystal violet. **B** Histogram showing the quantification of stained colonies. The data shown represent the means ± SD from three independent experiments. ns > 0.05, *p < 0.05, **p < 0.01 and ***p < 0.001, compared to control group.

PARP inhibitors are known to enhance glioma cell sensitivity to TMZ via the BER signaling pathway [26]. Our long-term cell survival assays revealed that combining QX302 with Olaparib significantly reduced the survival outcomes of T98G cells compared to monotherapy with either QX302 or Olaparib alone (P < 0.001) (Fig. 7A, B). This synergistic effect was likely due to Olaparib inhibiting the BER pathway, thereby preventing the repair of QX302-alkylated DNA, leading to increased cell death.

### Physicochemical ADME prediction of QX302

Lipophilicity (log P, log D) and the apparent permeability coefficient (Papp) are essential indicators of a compound’s potential for CNS penetration. To evaluate the ability of our compounds to penetrate the CNS, we utilized various predictive tools to assess their physicochemical properties. The recommended range for the octanol-water partition coefficient (log P) is between 2 and 5 [27], with an optimal range of 2 to 4 [28]. Similarly, the recommended values for the octanol-water LogP at pH 7.4 (log D) are between 2 and 3 [29]. Our predictions for both log P and log D values for QX302 fell within these ranges (Table 2), suggesting the analog has favorable BBB penetration capabilities.

**Table 2:** The CNS permeability of QX302.

Drug	A logP^1^	O logP^2^	R logP^3^	logPow^4^	logP^5^	logD^5^	Papp^5^ (cm/s)
QX302	4.03	3.59	3.66	3.932	2.464	2.461	2.171 × 10^−5^

1. Calculated with ALOGPS.

2. Calculated with OSIRIS Property Explorer.

3. Calculated with RPBS Web Portal.

4. Calculated with Online chemical database.

5. Calculated with ADMETlab 3.0.

Furthermore, we conducted an ADME analysis to predict the permeability of QX302 in Madin-Darby canine kidney (MDCK) cells. The Papp in MDCK cells, expressed in nm/s [30], was utilized as an indicator of the QX302’s potential permeability across the BBB. A higher Papp value correlates with greater cell permeability, with values exceeding 20 × 10^−6^ cm/s indicating high passive permeability. QX302 demonstrated Papp values above this threshold (Table 2), confirming its high passive permeability and reinforcing the validity of its predicted BBB penetration ability. These results collectively suggest that QX302 possesses the necessary physicochemical properties for efficient CNS penetration.

The Parallel Artificial Membrane Permeability Assay (PAMPA) is a commonly employed method in drug discovery for evaluating the BBB permeability of small molecules that has robustness and a high throughput ability [30]. In our investigation, we leveraged the NCATS Open Data ADME portal to assess QX302’s potential to traverse the BBB. QX302 exhibited moderate to high permeability according to the PAMPA model (Table 3). Collectively, these findings underscore the substantial lipophilicity and CNS-penetration capacity of QX302, consolidating its profile as promising for neuropharmacological applications.

**Table 3:** ADME@NCATS prediction of the pharmacokinetics and BBB permeability of QX302.

Model	Predicted class (Probability)	Prediction	Tanimoto similarity
HLC	0 (0.66)	stable	0.19
PAMPA	1 (0.78)	low or moderate permeability	0.29
PAMPA50	0 (0.98)	moderate or high permeability	0.23
PAMPABBB	0 (0.94)	moderate or high permeability	0.29
RLM	0 (0.66)	stable	0.29
Solubility	1 (0.94)	low solubility	0.29

## Discussion

TMZ is a key treatment for GBM, but its efficacy is limited by resistance mechanisms, including MGMT expression and MMR system inactivation. Developing TMZ-based agents that overcome these resistance pathways offers promising therapeutic potential. In this study, we synthesized a novel imidazotetrazine analog, QX302, through introducing phenylbutyrate nitrogen mustard at the C8 position of TMZ. QX302 demonstrated a reduction in cell viability in a dose- and time-dependent manner, with the highest cytotoxicity observed in U251 cells, exhibiting an IC50 value of 7.787 ± 0.32 μM. In T98G cells, characterized by high expression of MGMT, the IC50 was 86.13 ± 10.6 μM, indicating significantly enhanced cytotoxicity compared to TMZ [14]. QX302 exhibits significantly greater potency (approximately 2.37-fold) in MGMT-/MMR- glioma U87 cells compared to MGMT-/MMR+ glioma U251 cells, suggesting that MMR deficiency may play a role in conferring moderate resistance to QX302. In addition, the IC50 values for QX302 across various glioma cell lines (U251, U87, T98G, and HCT116) demonstrate that its efficacy is enhanced with prolonged exposure durations, underscoring the critical importance of extended treatment periods to optimize therapeutic outcomes.

To date, various TMZ derivatives, specifically modified at the C8 position, have been reported [15, 31–33]. For instance, the analog 2T-P400 connects two TMZ molecules via polyethylene glycol, thereby enhancing TMZ’s solubility and stability [31]. When the C8 carboxamide group of TMZ is substituted with a different moiety, a new derivative can be synthesized. For example, NEO212 forms a covalent bond between perilla alcohol and the C8 position of TMZ, thereby augmenting TMZ’s therapeutic efficacy [32]. Similarly, TMZ hexyl ester, which binds to the C8 site of TMZ, has been shown to improve both skin delivery and anti-tumor efficacy of TMZ [33]. Furthermore, additional C8-substituted derivatives, including C8-imidazolyl 377 and C8-methylimidazole 465 tetrazines, have been synthesized to counteract resistance mechanisms mediated by MGMT and MMR [15]. Unlike these TMZ derivatives, which mainly aim to improve alkylating potency or bioavailability to overcome resistance, QX302 acts through a dual mechanism: it induces both apoptosis and ferroptosis, independent of MGMT or MMR status. Importantly, QX302 not only triggers DNA damage but also activates ferroptosis, a non-apoptotic cell death pathway rarely engaged by conventional TMZ analogs. This unique mechanism may explain QX302’s superior efficacy in resistant GBM subtypes. Additionally, QX302 has shown activity at lower concentrations, potentially reducing off-target toxicity and improving its safety profile. These findings position QX302 as a promising candidate for addressing TMZ resistance through novel cell death modalities.

Comprehensive proteomics analysis revealed that QX302 alters the expression of proteins involved in nucleotide binding, chromatin organization, cell cycle regulation, and DNA replication/repair, suggesting a multifaceted mechanism distinct from TMZ-induced resistance pathways [34]. Functional assays, including colony formation and 3D sphere-forming models, demonstrated that QX302 more effectively inhibits glioma proliferation and tumor-like growth than TMZ. Flow cytometry showed that QX302 induces G2/M cell cycle arrest and apoptosis in a dose- and time-dependent manner. The elevated levels of cleaved caspase-9, caspase-7, caspase-3, and PARP further substantiated QX302’s role in activating the apoptotic pathway. The enhanced apoptotic effect observed at 72 h compared to 48 h highlights the time-dependent nature of QX302’s cytotoxicity.

Our findings demonstrate that QX302 effectively induces ferroptosis in GBM cells through the coordinated elevation of intracellular ROS, Fe²⁺ accumulation, and lipid peroxidation. Previous studies have demonstrated that TMZ promotes ferroptosis in glioblastoma cells via modulation of DMT1 expression, with its antitumor activity mediated through the Nrf2/HO-1 signaling pathway [35]. Compared to TMZ, QX302 exhibited a more robust effect across multiple GBM cell lines, as evidenced by higher ROS and Fe²⁺ levels and increased MDA content. In addition, QX302 downregulated key ferroptosis-regulatory genes SLC7A11 and ACSL3 at both the mRNA and protein levels, particularly showing a broader and stronger impact than TMZ. The suppression of SLC7A11, a critical component of the cystine/glutamate antiporter system, is of particular significance. SLC7A11 plays a crucial role in maintaining cellular redox homeostasis by regulating the influx of cysteine, which is essential for the synthesis of the antioxidant GSH. The downregulation of SLC7A11 results in decreased intracellular GSH levels, leading to oxidative stress and ferroptosis [36]. This mechanism is more pronounced in QX302-treated cells compared to TMZ, where SLC7A11 suppression is less pronounced, highlighting a distinct pathway through which QX302 induces ferroptosis in GBM cells. Furthermore, previous studies have reported that p53 can regulate ferroptosis through the inhibition of SLC7A11 expression [36]. Our study found that QX302 increases p53 expression, suggesting that QX302 may promote ferroptosis by activating p53, which in turn inhibits SLC7A11 expression. This adds another layer of complexity to the mechanism by which QX302 induces ferroptosis, potentially linking p53 activation to the downregulation of SLC7A11 and the subsequent oxidative stress-induced ferroptosis. These findings not only underscore the unique and potent ferroptosis-inducing mechanism of QX302 but also suggest that it may offer a more effective strategy for overcoming TMZ resistance, particularly in tumors exhibiting high SLC7A11 expression.

Further, we explored the DNA-damaging capabilities of QX302. Immunofluorescence assays showed increased γ-H2AX levels in QX302-treated glioma cells, indicating substantial DNA damage. The upregulation of p-ATM in response to QX302 treatment supports the involvement of DNA damage response pathways in the QX302’s activity. The in vitro alkylation assay demonstrated that QX302 effectively alkylated DNA at lower concentrations than TMZ and cisplatin, showcasing its potency as a DNA-alkylating agent. This potent DNA cross-linking ability likely contributes to QX302’s significant cytotoxic effects. Moreover, the combination of QX302 and Olaparib at low concentrations (1 μM) exhibited stronger synergistic cytotoxicity in T98G cells than the previously reported TMZ–Olaparib combination [37, 38]^0^, suggesting enhanced disruption of the BER pathway. Proteomic analysis revealed a moderate downregulation of PARP1 (fold change = 0.77, *p* = 0.07) upon QX302 treatment, while XRCC1 expression remained unchanged (fold change = 0.94, *p* = 0.66). These findings suggest that QX302 may partially impair BER initiation by reducing PARP1 levels without affecting the downstream repair scaffold. Given PARP1’s central role in sensing DNA strand breaks and recruiting XRCC1 to damage sites, even modest downregulation of PARP1 could significantly impair repair efficiency, especially under the additional stress of PARP inhibition by Olaparib. Additionally, our data demonstrate that QX302 induces ferroptosis in GBM cells by promoting ROS generation, Fe^2+^ accumulation, lipid peroxidation, and downregulation of SLC7A11 and ACSL3. This ferroptotic stress may further sensitize glioma cells to PARP inhibition. Previous studies have also shown that ferroptosis can synergize with Olaparib treatment by exacerbating oxidative damage [37, 38]^2^. Therefore, the synergy observed with QX302–Olaparib likely arises from a dual mechanism involving both suppression of DNA repair and enhanced ferroptosis induction. This mechanistic interplay supports the potential of QX302-Olaparib combination therapy in overcoming glioma resistance.

The physicochemical properties of QX302, including its log P and log D values, suggest it has favorable BBB-penetration capabilities. The high passive permeability demonstrated in the MDCK cell assays and PAMPA models provides further support for QX302’s potential for effective CNS penetration. These properties are critical for the development of therapeutic agents targeting brain tumors, underscoring QX302’s promise as an agent for neuropharmacological applications.

In conclusion, QX302 demonstrates potent anti-glioma activity by inducing DNA alkylation and cross-linking, even in MGMT-positive cells, leading to DNA damage, cell cycle arrest, ferroptosis and apoptosis. Its efficacy at lower concentrations relative to TMZ, coupled with its advantageous blood-brain barrier penetration properties, positions it as a promising candidate for glioma therapy. Despite the significant anti-tumor effects observed, further investigation is required to elucidate several aspects of QX302, including its water solubility and stability, in vivo metabolic pathways, and differential effects on various glioma subtypes. Additional studies are warranted to fully characterize its therapeutic potential and to investigate its efficacy in vivo.

## Materials and methods

### Biological materials and antibodies

The QX302 compound was synthesized as described previously [39]. TMZ and Olaparib were obtained from Leyan and Selleck Chemicals, respectively. Cisplatin (5 mM stock) was dissolved in H_2_O and stored at 4 °C, while TMZ (200 mM stock) and Olaparib (25 mM stock) were dissolved in dimethyl sulfoxide (DMSO) and stored at −20 °C.

Antibodies against GAPDH (5174S), caspase 3 (9662S), cleaved caspase 3 (9661S), caspase 7 (9492S), cleaved caspase 7 (9491S), caspase 9 (9502S), cleaved caspase 9 (9505S), cleaved PARP (5625S), CDK1 (CDC2, 9116S), p-CDK1 (p-CDC2, 9114S), cyclin B1 (12231 T), P53 (2524S), ATM (2873 T), p-ATM (4526 T), and γ-H2AX (80312S), and anti-rabbit IgG, HRP-linked antibody (7074S), and anti-mouse IgG, and horseradish peroxidase (HRP)-linked antibody (7076S) were procured from Cell Signaling Technology (Beverly, MA, USA). Goat anti-mouse IgG (H + L) cross-adsorbed secondary antibody, Alexa Fluor™ 488 (A-11001), was provided by ThermoFisher Scientific (Waltham, MA, USA).

### Cell culture

Human GBM U251, U87, T98G cell lines and the colon cancer HCT116 cell line were obtained from the Cell Bank of the Chinese Academy of Sciences (Shanghai, China). U87 cells were maintained in Dulbecco’s modified Eagle medium (Gibco, Waltham, MA, USA) supplemented with 10% fetal bovine serum (Ausbian, Shanghai, China). U251, T98G, and CT2A cell lines were cultured in Dulbecco’s modified Eagle medium/high glucose (HyClone, Marlborough, MA, USA) supplemented with 10% fetal bovine serum (PAN-Seratech GmbH Aidenbach). HCT116 cell lines were cultured in Roswell Park Memorial Institute (HyClone) medium supplemented with 10% fetal bovine serum (PAN, Aidenbach, Germany) and 1% penicillin/streptomycin (Gibco). All cultures were maintained at 37 °C with 5% carbon dioxide and 95% air in a humidified atmosphere. Cell passaging was performed by trypsinization of cells for 1–3 min at 37 °C, followed by neutralization with medium containing 10% FBS.

### Cell viability assay using CCK8

Cell viability was evaluated through a CCK-8 assay. Following digestion, resuspension, and enumeration, the T98G, U251, HCT116, and U87 cells were subsequently plated into 96-well plates at densities of 2500 cells/well for T98G, 4000 cells/well for U251, 6000 cells/well for HCT116, and 8000 cells/well for U87 in 100 μL of medium containing 10% FBS. Following a 24-h incubation period, the cells were exposed to varying concentrations of QX302 for the specified durations. Subsequently, 10 μL of CCK-8 reagent was introduced to each well containing 100 μL of complete medium, resulting in a total volume of 110 μL per well. The plates were subjected to an additional 2-h incubation period before absorbance readings were taken at 450 nm using an Infinite F50 Absorbance Microplate Reader (Berthold, Bad Wildbad, Germany).

### Colony formation assay

Prior to seeding into 6-well plates at the specified densities, cells were washed, trypsinized and counted. The plates were then incubated for 24 h in a cell incubator. Following this, the cells were treated with drugs at the indicated concentration and maintained for a duration of 10-14 days, with the medium being refreshed every 3 days. Upon completion of the treatment period, cells were washed with phosphate-buffered saline (PBS) and stained with 0.5% crystal violet solution containing 20% methanol. The stained colonies were subsequently analyzed using ImageJ software (Bethesda, MD, USA) for data processing and statistical analysis.

### Western blot analysis

Cell lysates were prepared and mixed with loading buffer (Macgene, Beijing, China), followed by denaturation at 95 °C for 10 min. The denatured proteins were separated using 8-15% sodium dodecyl sulfate-polyacrylamide gel electrophoresis and transferred onto polyvinylidene difluoride membranes (Millipore, Burlington, MA, USA). The membranes were blocked with 5% skim milk for 2 h at room temperature and incubated overnight with the primary antibody. Subsequently, the membranes were incubated with an HRP-conjugated secondary antibody for 2 h at room temperature. Chemiluminescent substrate (ThermoFisher, Waltham, MA, USA) was added, and the signal was detected using a Chemiluminescence Imager 5200 Multi (Tanon, Shanghai, China).

### Long-term cell survival assay

T98G cells were washed with PBS, dissociated using trypsin, counted, and plated into 6-well plates at a concentration of 3000 cells per well. After a 24-h incubation, the cells were treated with olaprib (3.125 μM) or DMSO for 30 min. The cells were subsequently exposed to TMZ alone (50 μM), QX302 alone (1 μM or 2 μM), QX302 in combination with olaprib (3.125 μM), or TMZ in combination with olaprib (3.125 μM) for 6 h. The culture medium was then refreshed, and the cells were cultured in standard medium for a duration of 10 days. Subsequent to this incubation period, the cells were rinsed with PBS, stained with 0.5% crystal violet solution supplemented with 20% methanol, and subjected to analysis using ImageJ software for data interpretation and statistical evaluation.

### Plasmid linearization assay

For the linearization reactions, 20 units of EcoRI-HF (New England Biolabs, Ipswich, MA, USA) were combined with 20 μg of 4361 bp pBR322 vector DNA in CutSmart buffer (New England Biolabs), pH = 7.9. The mixture was then incubated at 37 °C for 30 min to digest the DNA and obtain linear plasmids. The digested DNA was purified using a PCR gel recovery kit (Zymoclean, Irvine, CA, USA) and quantified using NanoDrop One (Thermofisher, Waltham, MA, USA). The purified DNA was stored at −20 °C until further in vitro DNA cross-linking analysis.

### In vitro DNA cross-linking assays

Linearized pBR322, prepared as described above, served as the substrate for the in vitro DNA cross-linking assays. For each experimental condition, 120 ng of linearized pBR322 DNA was incubated with 20 μL of the specified drug concentration. Drug stock solutions were prepared using DMSO to ensure a consistent 5% DMSO concentration in each reaction. Reactions were conducted in either double-distilled water or tris buffer (pH = 8.5). Incubation proceeded for 15 h at 37 °C. Following the reaction, DNA samples were stored at −80 °C until electrophoretic analysis. Gel electrophoresis was performed by mixing 10 μL (150 ng) of DNA solution with 2 μL of 6 × purple gel-loading dye (without SDS), which was loaded onto 1% agarose tris borate EDTA (TBE) gel. For denaturing gels, 10 μL (150 ng) of DNA solution was mixed with 30 μL of denaturation buffer (1% sodium hydroxide, 6% sucrose, and 0.4% bromophenol blue) on ice. The denatured DNA samples were then loaded onto a 1% agarose TBE gel and electrophoresed for 1.5 h at 70 V.

### Proteomics analysis

U251 cells were treated with QX302 (10 μM) or an equivalent volume of DMSO for 72 h. After treatment, the cells were lysed in 500 μL of RIPA buffer (Beyotime, Shanghai, China) to extract proteins. Total proteins were precipitated using precooled acetone, then denatured in 8 M urea in 0.1 M tris-HCl. Disulfide bonds were reduced with 10 mM DL-dithiothreitol and alkylated with 0.1 M iodoacetamide in the dark. Trypsin digestion was performed in 1 M urea at a ratio of 1/40 (enzyme/protein) at 37 °C. The resulting peptide mixture was desalted using a C18 column and dried under vacuum prior to liquid chromatography tandem mass spectrometry (LC-MS/MS) analysis. Samples were analyzed using a Triple TOF 6600 mass spectrometer (SCIEX) coupled with a Nano-ESI ion source. Mass spectrometry data were processed for total protein identification and quantification using ProteinPilot Software 5.0.1. GO enrichment and KEGG pathway analyses of differentially expressed proteins were conducted using Sangerbox (http://sangerbox.com).

### Cell apoptosis assays

For the apoptosis assays, the Annexin V-PE and RedNucleus II Apoptosis Kit (US Everbright, NY, USA) was employed. Cells were trypsinized and centrifuged according to the manufacturer’s instructions and washed and stained with Annexin V-PE and RedNucleus II in the dark at room temperature for 10 min. Apoptotic cells were then detected by flow cytometry (Becton, NJ, USA), and statistical analysis was performed using FlowJo (Ashland, OR, USA).

### Cell cycle analysis

For the cell cycle assays, U251 and U87 cells were trypsinized, centrifuged at 1000 rpm, and then washed with PBS. The cells were subsequently fixed in 75% ice-cold ethanol at 4 °C overnight. After fixation, the cells were stained in the dark with propidium iodide staining solution (Yeasen, Shanghai, China) for 30 min. The cell cycle was analyzed by flow cytometry, and statistical analysis was conducted using FlowJo.

### Tumor cell spheroid assay

For the tumor cell spheroid assay, U87 cells were digested, centrifuged, counted, and then plated at a density of 2000 cells per well on a low-adsorption 96-well plate. After 2 days of normal cell growth, the cells were treated with QX302 (10, 20, or 40 μM) for 6 days, with DMSO serving as the negative control and TMZ (400, 800, or 1600 μM) as the positive control. Following treatment, cells were stained using the Live/Dead Cell Double Staining Kit (Bestbio, Shanghai, China), and images were captured using a fluorescence microscope (Zeiss, Oberkochen, Germany) equipped for 488 nm detection. Statistical analysis was performed using ImageJ and GraphPad Prism 6.02 software (San Diego, CA, USA).

### Immunofluorescence

Cells were digested with trypsin, centrifuged, counted, and then cultured at a density of 1 × 10^5^ (U251, U87) and 5 × 10^4^ (T98G) cells in 24-well plates with round slides. After 24 h, the cells were treated with QX302, DMSO (negative control), or TMZ (positive control) for 72 h, followed by fixation with 4% paraformaldehyde for 15 min. Subsequently, the cells were treated with 0.5% Triton X-100 for 15 min, blocked with 5% bovine serum albumin for 1 h at room temperature, and incubated overnight with anti-γ-H2AX (1:500) primary antibody. Following this, the cells were reacted in the dark with anti-mouse IgG (H + L) secondary antibody. Nuclei were stained with 4’,6-diamidino-2-phenylindole (DAPI). Image acquisition was performed with a laser confocal microscope (ZEISS-LSM880), and fluorescence intensity was analyzed by ImageJ software.

### Measurement of reactive oxygen species (ROS) and Fe^2+^ levels>

Flow cytometry was used to assess ROS and Fe²⁺ levels in QX302-treated GBM cells. For ROS detection, cells were stained with 10 µM DCFH-DA (Invitrogen, CA, USA) for 30 min at 37 °C in the dark, followed by two washes with warm PBS and trypsinization. The cells were then resuspended in PBS and immediately analyzed by flow cytometry using a BD C6 cytometer within 20 min. For Fe²⁺ detection, cells were stained with 1 µM FerroOrange (DOJINDO, Shanghai, China) for 30 min at 37 °C in the dark, and fluorescence was measured by flow cytometry and analyzed using C6 software.

### Measurement of malondialdehyde (MDA) levels

Intracellular concentrations of MDA were detected by an MDA assay kit (Jiancheng, Nanjing, China). The detailed measurement was conducted according to the kit instructions.

### Physicochemical of Absorption, Distribution, Metabolism, Excretion (ADME) prediction

Computational evaluation through ALOGPS (https://vcclab.org/lab/alogps/), OSIRIS Property Explorer (https://www.organic-chemistry.org/prog/peo/cLogP.html), RPBS Web Portal (https://mobyle.rpbs.univ-paris-diderot.fr/cgi-bin/portal.py?form=admetox), Online chemical database (https://ochem.eu/home/show.do), ADMETlab 3.0 (https://admetlab3.scbdd.com) and ADME@NCATS (https://opendata.ncats.nih.gov/adme/home) online platforms, the Lipophilicity (log P, log D), the apparent permeability coefficient (Papp) and BBB permeability of the QX302 were predicted.

### Statistical analyses

Statistical analysis was performed using GraphPad Prism 6.02. Student’s t-tests were employed to compare differences between two groups. A p-value less than 0.05 was considered statistically significant for all tests.

## Supplementary information

Supplementary Material

## Data Availability

The data generated in this study are available upon request from the corresponding author.
