# Supplementary material for: Novel imidazotetrazine derivatives overcome temozolomide resistance in glioblastoma by inducing ferroptosis and apoptosis
Source: Cell Death Discov. 2026 Jan 9;12:14. doi: 10.1038/s41420-025-02857-3 (PMC12789667; doi:10.1038/s41420-025-02857-3)
Supplement: Supplementary file 1 — Supplementary Material [file 41420_2025_2857_MOESM1_ESM.docx]

**
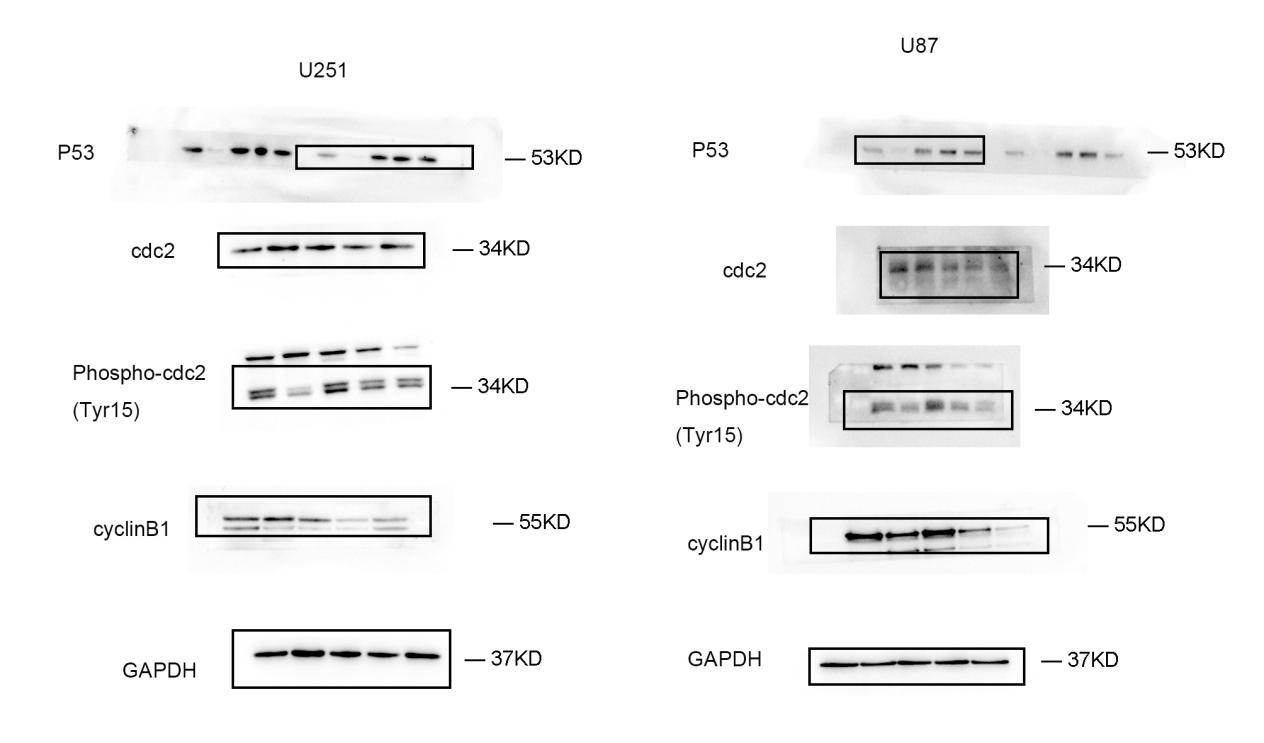
**

**Uncropped images corresponding to those shown in Figure 3 F**

**
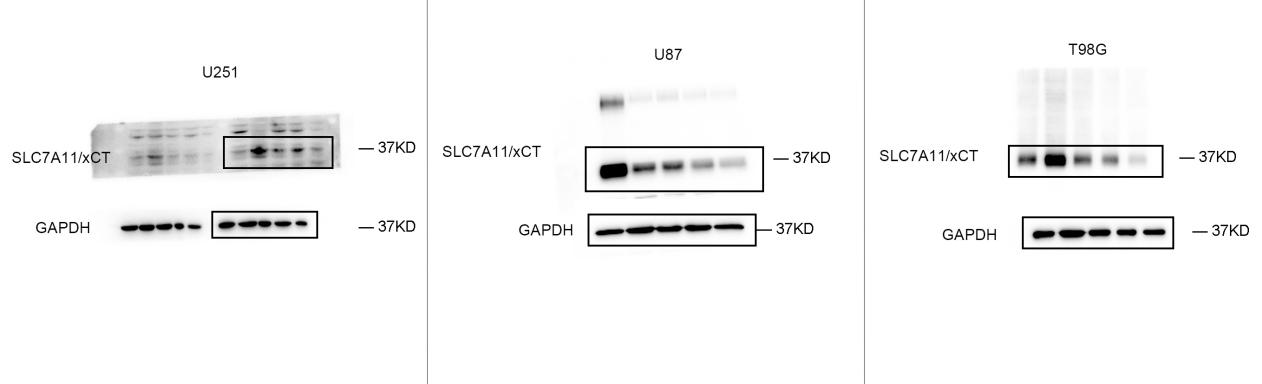
**

**Uncropped images corresponding to those shown in Figure 4 F**

**
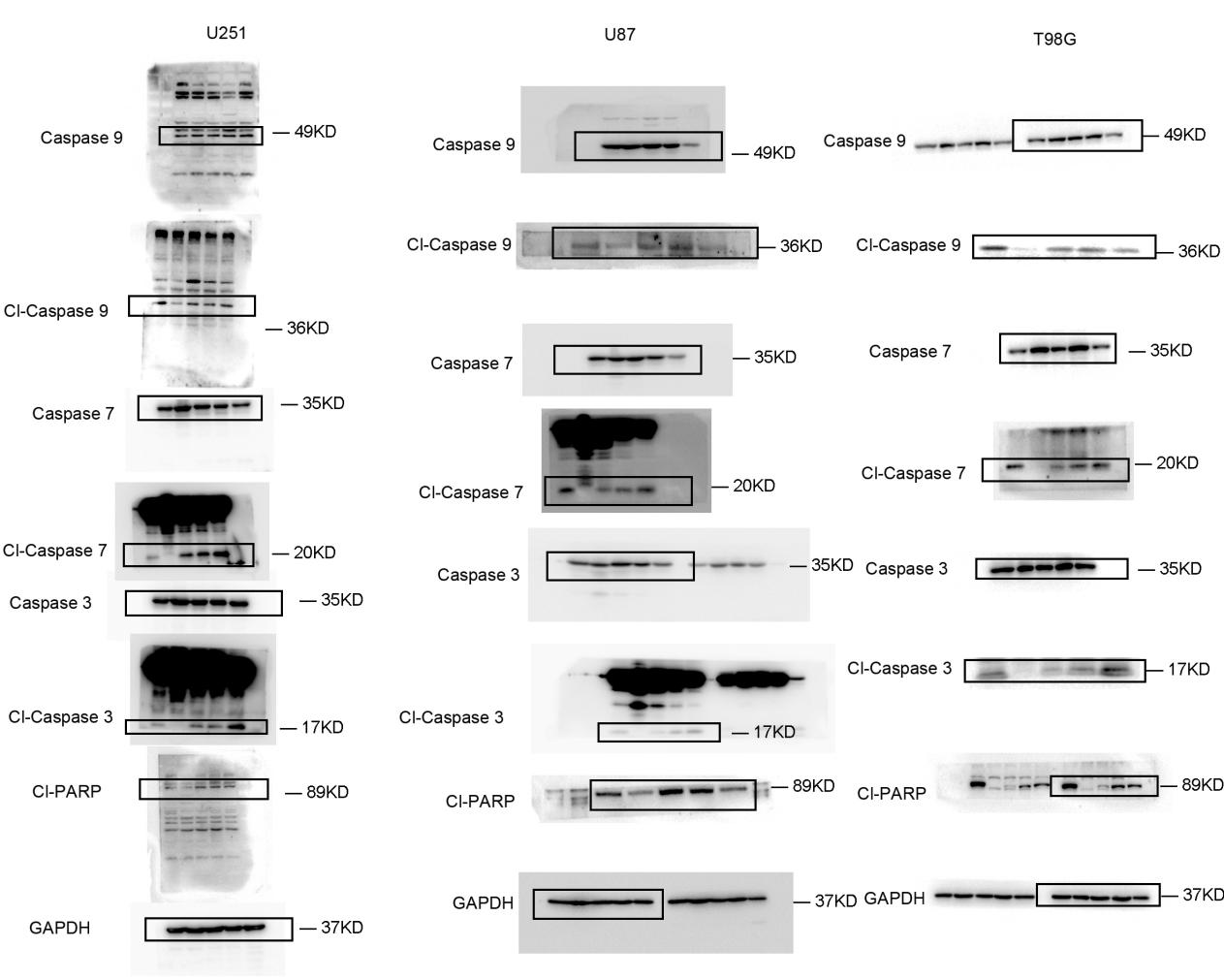
**

**Uncropped images corresponding to those shown in Figure 5 D**

**
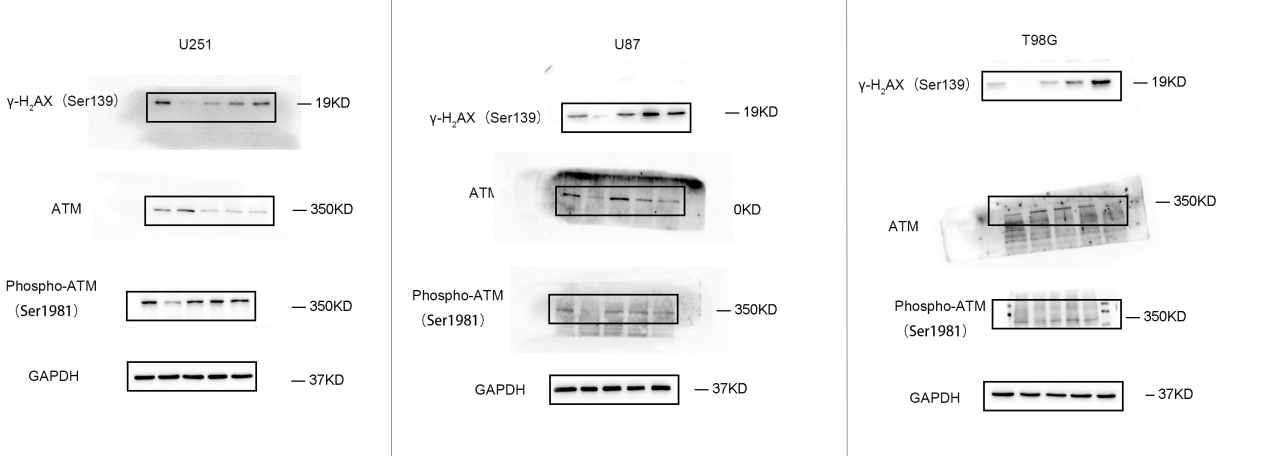
**

**Uncropped images corresponding to those shown in Figure 6 D**
